# Economic, environmental, and social sustainability in scheduled hybrid office work

**DOI:** 10.1093/annweh/wxag010

**Published:** 2026-03-05

**Authors:** Marina Heiden, Alexander Wejskog, Gunnar Bergström, Svend Erik Mathiassen, David Hallman, Sandra Sjöberg

**Affiliations:** Centre for Musculoskeletal Research, Department of Occupational Health, Psychology and Sports Science, Faculty of Health and Occupational Studies, University of Gävle, 801 76 Gävle, Sweden; Centre for Musculoskeletal Research, Department of Occupational Health, Psychology and Sports Science, Faculty of Health and Occupational Studies, University of Gävle, 801 76 Gävle, Sweden; Centre for Musculoskeletal Research, Department of Occupational Health, Psychology and Sports Science, Faculty of Health and Occupational Studies, University of Gävle, 801 76 Gävle, Sweden; Unit of Intervention and Implementation Research for Worker Health, Institute of Environmental Medicine, Karolinska Institutet, Box 210, 171 77 Stockholm, Sweden; Centre for Musculoskeletal Research, Department of Occupational Health, Psychology and Sports Science, Faculty of Health and Occupational Studies, University of Gävle, 801 76 Gävle, Sweden; Centre for Musculoskeletal Research, Department of Occupational Health, Psychology and Sports Science, Faculty of Health and Occupational Studies, University of Gävle, 801 76 Gävle, Sweden; Department of Community Development, The Municipality of Borlänge, 781 81 Borlänge, Sweden

**Keywords:** telework, economic sustainability, environmental sustainability, social sustainability, intervention

## Abstract

We investigated effects on economic (cost savings), environmental (CO_2_ emission), and social sustainability (factors reflecting a positive psychosocial environment at work) of a 6-mo intervention from November 2021 through April 2022, during which employees were offered scheduled telework in a municipality in Sweden. Economic and environmental sustainability were estimated by the municipality whereas social sustainability was assessed by the researchers. Using analysis of covariance, we examined outcomes in 3 groups, ie scheduled teleworkers (*n* = 36), non-scheduled teleworkers (*n* = 55), and office workers (*n* = 11). The results indicated that scheduled telework resulted in reduced costs since unutilized premises could be disposed, and less CO_2_ emission due to a combination of reduced commuting time, lower energy use, and less need to construct new buildings. However, the 3 groups did not differ significantly regarding social sustainability. The results support the notion that telework can lead to positive outcomes in terms of decreased premises costs and environmental footprint, without deteriorating social sustainability.

What's important about this paper Telework was adopted to a large extent during the COVID-19 pandemic, and it will likely remain in post-pandemic times. This intervention study investigated if scheduled telework contributes to economic, environmental, and social sustainability at work and found that it improved economic and environmental sustainability without compromising social sustainability.

## Introduction

Hybrid work, ie switching between when, where and how work is performed (Lauring and Jonasson 2025), has caused organizations to consider the use of office spaces. Studies have shown that reduced occupancy of office buildings can lead to less energy consumption and emissions if the buildings are equipped to adapt to changing occupancy levels (Mantesi et al 2022; Sepanta et al 2024). Furthermore, more efficient use of the office space may contribute to cost savings; for example, with staff teleworking 50% of the time, less offices would be needed for the staff and the office rent could be reduced accordingly. A major challenge for sustainable use of office space, however, is predicting how it will be used (Motuzienė et al 2022).

In September 2020, when Swedes were strongly recommended to work from home due to the Covid-19 pandemic (Ludvigsson 2023), the management in a municipality in central Sweden asked the local urban development sector to investigate the possibility for office workers to telework on a regular basis. A reason for the initiative was that employees perceived telework positively and wished to continue teleworking regularly even after the pandemic. The municipality also believed that the initiative would allow disposing of unutilized premises, thus reducing costs, and improve the organization's environmental footprint by decreasing CO_2_ emission caused by less energy consumption and reduced commuting to the office. Furthermore, to facilitate a sustainable social climate in the working teams, the municipality also supported physical co-working by initiating a fixed weekly telework schedule for the employees.

The aim of this study was to evaluate the effects of this new way of working in the organization during a 6-mo trial period. The outcomes of interest were CO_2_ emission as an indicator of environmental sustainability, cost savings for office premises as an indicator of economic sustainability, and several aspects of work environment and health as indicators of social sustainability (Salome Correia 2019).

## Methods

### Intervention

**Figure 1:**
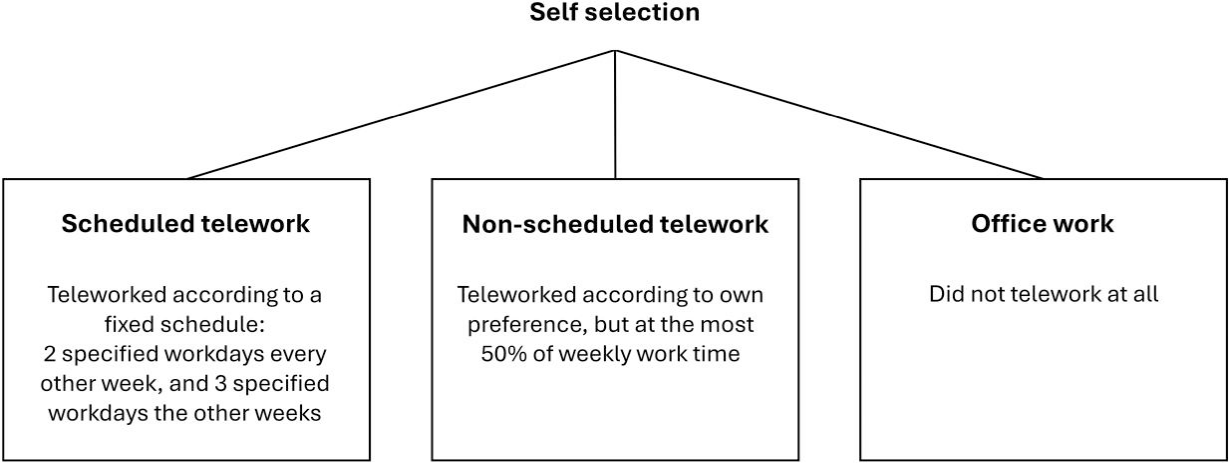
Self-selected intervention groups in the study.

Five departments of the urban development sector of the municipality were invited to sign up to telework 50% of their work time on a fixed schedule for 6 mo. Employees volunteering to telework on a fixed schedule (Scheduled telework; referred to as the ST group) had to work 50% from the office every week and were assigned a fixed office space. Employees who did not volunteer to telework according to the schedule chose to either telework at most 50% according to their preference during each work week (non-scheduled telework; NST group) or work entirely from the office (office work; OW group) (Fig. 1). For the ST group, colleagues were scheduled to work at the office on the same days, thus promoting social community at work. In contrast, employees in the NST group could not be sure that their colleagues were present. The intervention took place between November 2021 and April 2022 with an unexpected pause of 10 wk due to sharpened pandemic restrictions.

### Data collection and participants

The population comprised 65, 142, and 25 employees at 5 departments in the ST, NST, and OW groups, respectively. At baseline, 157 of the 232 employees (68%) responded to a questionnaire. The respondents were of similar age to non-respondents (mean age 45.9 versus 43.3 yr) but more likely to be female (73% versus 51% women). At follow-up, the questionnaire was answered by 127 employees (55%). The final sample comprised only those participants who had answered both questionnaires (*n* = 102). Their mean age was 47.1 yr, and 75% were women.

Economic sustainability was estimated by the municipality by approximating savings in rent for the premises in the ST group, where each office was used by 2 employees instead of one. Environmental sustainability was estimated, also by the municipality, in 3 ways. First, CO_2_ emissions when commuting by car were estimated using a template from The Swedish Environmental Protection Agency. The estimates were based on the number of employees in the ST group normally commuting by car, their average distance to work, and the number of trips needed when teleworking 50% compared to not teleworking at all. Second, CO_2_ savings due to reduced use of premises in the ST group were calculated based on the building's average energy use for heating. Third, CO_2_ savings due to less needs for construction of offices to accommodate all staff were estimated based on current figures from the Swedish Environmental Protection Agency.

Social sustainability was assessed by the researchers on basis of questions about the psychosocial work environment in COPSOQ-III (Berthelsen et al. 2020), the WHO-5 well-being index (Topp et al. 2015), and a question regarding self-assessed productivity. The questionnaire was distributed online using Qualtrics (Qualtrics, Provo, Utah) before (baseline) and immediately after the 6-mo intervention period (follow-up). The study was ethically approved by the Swedish Ethical Review Authority (Ref. no. 2019-06220).

### Statistical analysis

Characteristics of the participants are presented as proportions, means, and standard deviations (SD). Indicators of social sustainability are presented as means, SD, and 95% confidence intervals for baseline data and for the change to follow-up. Change scores from baseline to follow-up were compared between the 3 groups using analysis of covariance (ANCOVA), with adjustment for age, sex, and the baseline value of each specific outcome. The covariates were selected in view of the size of the dataset and previous studies on telework (Heiden et al 2023; Januario et al 2025). All analyses were performed by the researchers without involving the municipality in IBM SPSS statistics version 29 for Windows (IBM Corp., Armonk, NY, USA), with the level of significance set at *P* < 0.05. Model assumptions were checked using homogeneity of variance tests, lack-of-fit tests, and standard graphical procedures.

## Results

The final sample (*n* = 102) consisted of 36, 55, and 11 employees in the ST, NST, and OW groups, respectively. Their mean (SD) age was 44.8 (10.3), 49.4 (10.8), and 43.4 (12.5) yr. The 3 groups comprised 78%, 71%, and 82% women, respectively.

The estimated annual savings for premises and CO_2_ in the ST group, both per employee, are shown in Table 1. Table 1 also shows baseline and change scores to follow-up for indicators of social sustainability. The ANCOVA showed that none of these scores differed significantly between the 3 groups (Table 1).

**Table 1:** Indicators of economic, environmental and social sustainability at baseline and their change during the intervention period (follow-up) among participants with ST (*n* = 36), NST (*n* = 55), and OW (*n* = 11).

**Economic sustainability**	
Savings for the premises in the ST group	23,000 SEK (corresponding to €2,000) per employee and year
**Environmental sustainability**	
CO_2_ savings due to reduced commuting by car in the ST group	0.35 to 0.50 ton per employee and year
CO_2_ savings due to reduced energy use for heating in the ST group	0.18 ton per employee and year
CO_2_ savings due to less need for construction of offices	9.0 ton per employee and year

	Social sustainability							
	Baseline			Follow-up			Partial η^2^	*P*
	Mean (SD)			Mean difference from baseline^a^ (95% CI)				
	ST	NST	OW	ST	NST	OW		
Productivity (0 to 10)	8.8 (1.5)	9.0 (1.5)	7.5 (2.0)	−0.3 [−0.7, 0.2]	0 [−0.3, 0.3]	−0.3 [−1.1, 0.4]	0.01	0.51
Sense of community at work (0 to 100)	78.5 (15.1)	85.0 (11.4)	75.8 (10.2)	−3.5 [−8.6, 1.5]	−2.7 [−6.8, 1.4]	−9.5 [−18.6, 0.4]	0.02	0.43
Social support from supervisor (0 to 100)	81.9 (20.4)	74.1 (28.9)	75.0 (15.8)	−2.1 [−9.5, 5.2]	−2.5 [−8.5, 3.4]	−13.3 [−27.2, 0.6]	0.03	0.29
Social support from colleagues (0 to 100)	81.9 (18.5)	80.5 (19.7)	79.5 (18.8)	−5.3 [−11.1, 0.5]	0.4 [−4.3, 5.1]	−0.3 [−10.8, 10.2]	0.03	0.24
Well-being (0 to 100)	57.7 (18.6)	60.5 (18.9)	49.8 (18.3)	1.0 [−3.8, 5.9]	1.3 [−2.7, 5.2]	−3.9 [−12.6, 4.8]	0.01	0.54

CI, confidence interval.

^a^Value at baseline subtracted from the follow-up, ie negative values indicate a reduction over time. The value is not adjusted for covariates.

*P*: Significance of difference between the 3 groups in the change during the intervention period; ANCOVA adjusted for age, sex, and the baseline value of the outcome.

In the ANCOVAs, Levene's test showed that equal variance across the categories of respondents could be assumed for each outcome (*P* > 0.170). Lack-of-fit was indicated in the analysis of social support from colleagues. Attempts to transform the variable did not improve the model fit or change the results. When inspected, the residuals did not show large deviations from normality (skewness: 0.43 to 1.04; kurtosis: 0.26 to 1.89).

## Discussion

To our knowledge, this is the first study to investigate effects of an organizational intervention addressing ST, NST, and OW on economic, environmental, and social sustainability. Our main findings suggest that introducing ST may promote both economic (ie reduced premises costs) and environmental sustainability (ie lower CO_2_ emissions due to reduced commuting time, lower energy use, and less need to construct new buildings), compared to OW. However, our results also indicated a small and generally negative effect over time on the social sustainability indicators, mainly in the OW group, even if the difference between groups was not significant. This may suggest that the presence of colleagues when working (which was organized in the ST and OW groups) was less important for the work environment and health indicators investigated in the study. It is also possible that effects of teleworking up to 50% on psychosocial work environment, well-being, and productivity are smaller than expected. In this study, the self-selection of participants into groups, which improved the match between telework conditions and individual preference, may have contributed to better ratings across all groups than if a randomized controlled study design had been used (Bloom et al 2024). Since the study was performed at the end of the Covid-19 pandemic, there may be context-specific factors relating to the participants' experience of their work environment and health that we have not controlled for.

The findings are not in agreement with de Vries et al (2019), who found that public servants experience greater professional isolation when teleworking from home. However, they also found that the quality of leadership mitigated the effect of telework on professional isolation, which was not investigated in this study. According to a systematic review by Vleeshouwers et al (2022), the evidence of association between teleworking from home and professional isolation is low. In addition, Vleeshouwers et al (2022) found low quality of evidence for a relationship between teleworking from home and productivity, with productivity being measured in different ways. Mutiganda et al (2022) systematically reviewed studies on telework and performance and showed that the selection of performance metric may result in different findings. While self-rated performance was generally higher among teleworking employees than those working in the ordinary workplace, objective organizational performance indicators showed more promising results in homogeneous samples with specified work tasks. In this study, however, productivity was self-assessed and of similar magnitude in the ST, NST, and OW groups at baseline as well as follow-up.

Our study suffered several limitations. While cost savings on rent were only made in the ST group, since the OW and NST groups kept their offices throughout the study period, we did not know the extent of teleworking in the NST group, and therefore no estimates of CO_2_ savings are presented for this group. The study was performed as part of the municipality's adaptation to new ways of working after the Covid-19 pandemic, and the groups were self-selected, which prevented control of group size and may have led to potential bias due to group preference. Furthermore, at the baseline measurement of social sustainability indicators, respondents were more likely to be female than non-respondents. Thus, the generalizability of the findings may be limited. As the estimates of CO_2_ emissions were based on yearly averages, they may underestimate the effect on environmental sustainability during the study period (November to April). Also, we cannot deduce whether reduced costs and emissions for the municipality were offset by increased costs and emissions elsewhere, since we did not gather information about the employees' residences or potential changes in daily routines resulting from participating in the study. We encourage future research and practice to evaluate similar interventions by adapting a randomized controlled design and including a process evaluation that considers qualitative insights from both management and employees. Studies may also benefit from addressing sustainability indicators in all the different areas where work is performed.

## Conclusion

Scheduled teleworking for 50% working time was successful in increasing economic and environmental sustainability without notable effects on social sustainability. From the employer's perspective, this may offer an attractive opportunity to reduce the organization's environmental footprint. We recommend further studies examining these associations, with particular emphasis on how to organize telework, and incorporating long-term follow-up with qualitative feedback from managers and employees.

## Data Availability

The data underlying this article will be shared on reasonable request to the corresponding author.
